# Alteration in *CatSper*1 and 2 genes expression, sperm parameters and testis histology in varicocelized rats

**Published:** 2018-03

**Authors:** Maryam Zohour Soleimani, Farideh Jalali Mashayekhi, Morteza Mousavi Hasanzade, Maryam Baazm

**Affiliations:** 1 *Students Research Committee, Arak University of Medical Sciences, Arak, Iran.*; 2 *Department of Biochemistry and Genetics, School of Medicine, Arak University of Medical Sciences, Arak, Iran.*; 3 *Department of Anatomy, School of Medicine, Arak University of Medical Sciences, Arak, Iran.*

**Keywords:** Varicocele, CatSper, Sperm parameters, Rat

## Abstract

**Background:**

*CatSper* gene, a member of cation channel sperm family, has an essential role in sperm motility and male fertility. Following varicocele, sperm parameters especially sperm movement decreases. For this reason, we hypothesized that *CatSper* gene expression might be reduced after varicocele induction in an animal model.

**Objective:**

The aim of this study was to evaluate the expression of *CatSper* 1 and 2 genes, sperm parameters and testis histology following varicocele induction**.**

**Materials and Methods:**

A total of 30 Wistar male rats were randomly divided into three following groups (n=10/ each): control, sham, and varicocele group. Experimental varicocele was induced by partial ligation of the left renal vein. The epididymal sperm parameters, *CatSper*1 and 2 genes expression, and testes histology were studied two months after varicocele induction.

**Results:**

Our results revealed that motility (32.73±16.14%), morphology (48.80±17%) and viability (31.23±9.82%) of sperms significantly reduced following varicocele induction. In addition, we showed a significant decrease in the number of spermatogonia (43.63±5.31) and seminiferous tubules diameters (190.51±19.23 mm) in experimental varicocele rats. The level of *CatSper*1 and 2 genes expression evaluated using real-time polymerase chain reaction was significantly downregulated 2 months after varicocele induction.

**Conclusion:**

Our data indicated that experimental varicocele has deleterious effects on sperm parameters, testis structure as well as the expression of *CatSper* 1 and 2 genes.

## Introduction

Varicocele is the most common surgical treatable cause of male infertility (1). The incidence of varicocele is about 15% of the total population of men (2). The varicocele occurs in both testis; but because of the differences in the anatomical structure of testicular vein in the left and right sides, varicocele in the left side is more common (3). During varicocele, enlargement occurs in the testicular vein plexus (pampiniform plexus) that consists of internal spermatic and cremasteric veins (1, 4). Most of the time, varicocele causes no problem and is harmless, but in some patients, it can damage testis tissue and induce infertility (5). The mechanisms are involved in the pathogenesis of varicocele is unclear; but various hypotheses suggested by previous researchers including increasing in the testicular temperature, venous stasis, accumulation of CO_2_, nitric oxide and reactive oxygen spices (ROS), autoimmunity, and retrograde flow of toxic metabolite from adrenal gland (6-9). These pathological events impair normal spermatogenesis, decrease semen quality and could affect sperm parameters such as sperm count, motility and morphology (10, 11).

Sperm motility is one of the critical steps in fertilization. Intracellular Ca+2 concentration plays an important role not only in sperm movement (12) but also in sperm capacitation and egg penetration (12, 13). There are different channels for entering Ca+2 into the sperm cytoplasm. *CatSper*, a cation channel of sperm, is a pH gated channel and consists of four pore-forming proteins (CatSper1-4) (14, 15). The alkaline environment of the female reproduction tract activates the CatSper channels. The opening of these channels increases the intracellular Ca+2 concentration and consequently hyper-activates sperm (14). Hyper-activation, a type of asymmetric motility, is vital for fertility (16, 17) and impairment in the function of the CatSper channels and its related genes will disturb fertility in both human and animals (18, 19).

Previous studies suggested that the expression of *CatSper* gene in testis tissue is affected by some factors (20-22). Aging is a process that can decrease sperm parameters. Some researchers showed that in aging mice both sperm motility and *CatSper* gene expression decreased and by using Escanbil (Calligonum) extract the expression of *CatSper* 2 and 4 and sperm motility increased (20). In addition, it is believed that the reduced sperm motility in the spinal cord injury animal model might be due to the decreasing *CatSper* 1 and 2 expression levels (21). The expression of *CatSper* genes could be affected by Bisphenol A (22) and Kerack (23). These two materials by impairing spermatogenesis decrease sperm motility and the expression level of *CatSper*. Previous studied showed that there is a correlation between sperm parameters and *CatSper* gene expression in the testis of Iranian Kerack-addicted mouse (23). On the base of these studies, it was assumed that the reducing in sperm motility during varicocele may be related to the changes of expression levels of *CatSper* genes. 

Therefore, in this study for the first time, we aimed to investigate whether *CatSper* gene expression is affected by varicocele.

## Materials and methods

**Animals**

In vivo experiments were performed in 14 wk adult male Wistar rats (200-250 gr, Pasteur, Iran). Animals were housed at 24^o^C under controlled conditions with free access to water and food.

Animals were randomly divided into the following groups (n=10/ each): Control, sham operation and left experimental varicocele (LEV) induction.

**Surgical procedure**

Rats were anaesthetized with intraperitoneal (IP) injection of 100 mg/kg ketamine and 10 mg/kg xylozine (both from Alfasan, Iran) (24). After shaving and cleaning the surgical area, a midline incision was performed. For partial ligation in left renal vein, a 0.85 mm wire was placed parallel to the left renal vein and a 4-0 silk suture was used for ligation around the wire and left renal vein proximal to the inferior vena cava. Then the wire was carefully removed and the abdominal wall was sutured (10). In the sham group, the similar procedure except for the partial ligation of the left renal vein was done.

**Sperm analysis: motility, count, morphology, and viability**

Two months after varicocele induction, the left cauda epididymis was carefully removed and placed in 5 ml of phosphate buffer saline solution (PBS; Sigma, Germany). After mincing the cauda, the acquired suspension was incubated at 37^o^C in 5% CO_2_ for 30 min. Next, one drop of sperm suspension was placed in a Neubauer chamber and covered by the cover slide. Then, the percentage of motile sperm and sperm count was evaluated under a light microscope (for the sperm motility evaluation a ×400 magnification was used). The sperm count was expressed as ×10^6^/ml.

For sperm morphology evaluation Papanicolaou staining was used and one hundred sperm from different fields were counted to determine the morphological abnormalities (25). 

Eosin-B (Merck, Germany) staining was used for sperm viability analysis. According to this staining, the dead sperm was red and live sperm stayed unstained (26). One hundred sperm cells were counted for each sperm sample and were expressed as the percentage of viable sperm. In each sperm smear one hundred sperms were analyzed and the percentage of white sperms was expressed as viable sperm.

**Testicular histology**

To examine the testicular histology, testis tissue was fixed by 4% paraformaldehyde (Sigma, Germany) and after histological processing; the 7-μm thickness sections were prepared and stained with hematoxylin-eosin (Merck, Germany) (21). In seminiferous tubules from each mouse (20 microscopic fields), the number of spermatogonia was counted (27) and seminiferous diameter was measured from basement membrane to basement membrane by Image Tools analysis software (28).

**RNA isolation and cDNA synthesis**

After sampling the expression of *CatSper* 1 and 2 genes and CycloA (as an internal control) in all groups was studied by quantitative reverse transcriptase polymerase chain reaction (qRT-PCR). Total RNA was extracted using peqGold RNA TriFast (PeqLab, Germany) according to the manufacturer's instructions. The RNA pellet was dissolved in diethylpyrocarbonate-treated water (DEPC treated water; SinaClon, Iran) and quantified spectrophotometrically at 260 nm wavelength. The integrity of the extracted total RNA was assessed by agarose gel electrophoresis and verified by the presence of the 28S and 18S rRNA bands. Immediately after RNA preparation, 2 μg of total RNA was used for cDNA synthesis in a total volume of 20 μL by using RevertAid™ First Strand cDNA Synthesis Kit (Aryatous, Iran). The cDNA was stored at -70^o^C until use.

We confirmed RNA integrity by electrophoresing the extracted RNA on agarose gel and determining the 28S and 18S rRNA bands. 2µg of total RNA was used for cDNA synthesis according to kit protocol (Aryatous, Iran) and stored at -70^o^C for future studies. 

**Quantitative RT-PCR**

qRT-PCR was carried out using the Life Cycler Real-time PCR (Roche, USA). qRT-PCR was performed in a total volume of 20 μL containing 2 μL of cDNA (5-fold diluted), 0.5 μL of 5 mmol/l solutions of each of the forward and reverse primers, and 10 μL of 2x SYBR green DNA PCR Master Mix (Yekta Tajhiz Azma, Iran). Each sample was loaded in duplicate. Primer sequences for real-time PCR was: *CatSper*1, 5'-TCT TGG AGC GAT GAG GAC and rev 5'- GAC GAT TGT GTT CAG GCA; *CatSper* 2, 5'-TGG TTG TTG CTT GGT TCC and rev 5'-TTC CTT GAC TGG TTC CTC T; Cyclo A, 5'-GGC AAA TGC TGG ACC AAA CAC and rev 5'- TTA GAG TTG TCC ACA GTC GGA GAT G (for normalization in real-time PCR). Melt curve analysis was performed after each run to check for the presence of non-specific PCR products and primer dimers. The expression ratio was calculated using a relative formula based on the comparative CT method (ΔΔCT). 

**Ethical consideration**

Research and animal care were approved by the Ethics Committee of Arak University of Medical Sciences. 

**Statistical analysis**

The results are expressed as the mean±SD. The statistical significance of the mean values was determined by one-way analysis of variance (ANOVA) followed by a Tukey post-test with p≤0.05 as the statistically significant criterion.

## Results

**Varicocele changed sperm parameters**

**Figure 1:**
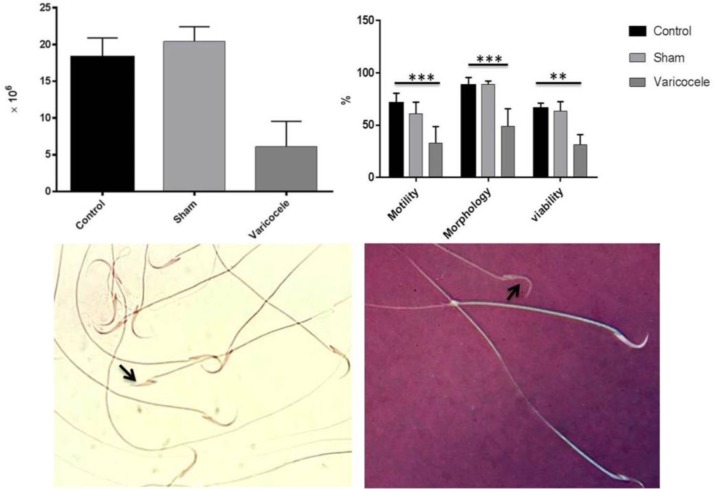
Sperm parameters including count, motility, morphology and viability are presented in different groups. Note the decline of sperm parameters in varicocele-induced animals compared to sham and control (A and B). Sperms obtained from the tail of epididymis (C). Abnormal morphology of sperm head (left, arrow). After Eosin B staining, dead sperms appeared red (right, arrow).

According to our results, in the varicocele group the sperm count was reduced (6.12×10^6^ ±3.41) though this decrease was not statistically significant in comparision to the other groups (control: 18.2×10^6^ ±2.6 and sham: 20.4×10^6^±1.91) (p=0.06) (Figure-1A). In all three groups, we analyzed the sperm motility. Our data showed that varicocele significantly decreased (32.7±16.1%) sperm motility 2 months after left renal vein ligation (p≤0.001) (Figure-1B). The sperm morphology was investigated after papanicolaou staining. The normal morphology was significantly decreased (48.80±17%) in the varicocele group in comparision to the control (88.2±7.5%) and sham (89±3.3%) operated groups (p=<0.001) (Figure-1B). Figure-1C shows an abnormal sperm in the varicocelized rats. The results acquired from eosin B staining showed a significant reduction in the sperm viability of the varicocele group (31.23±9.82%) in comparision with other animal groups in this study (control: 66±5.22, sham: 63.7±8.81) (p=0.007) (Figure-1B). In this staining dead sperms allow eosin B to enter cytoplasm and appear red (Figure-1C).

**Varicocele changed normal testis histology**

**Figure 2:**
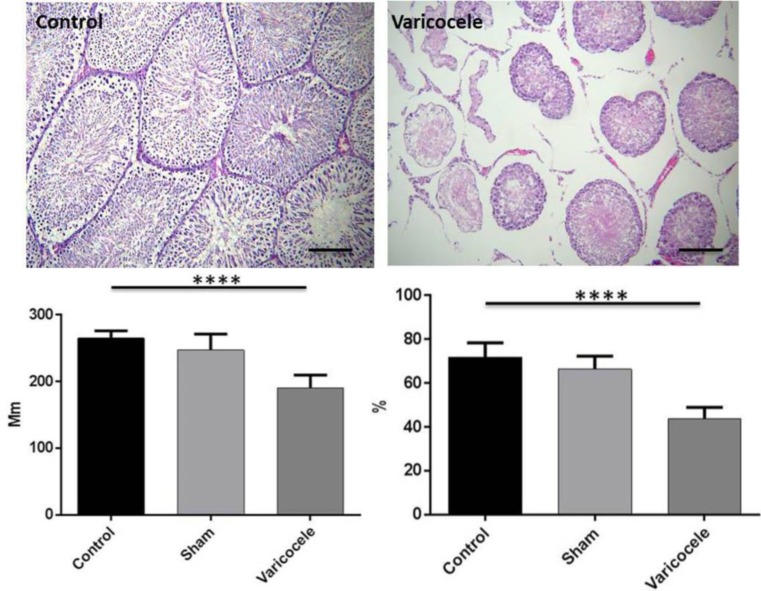
Testis histological sections were investigated under a light microscope (A). Note the significant decrease in seminiferous tubules diameter (B) and a number of spermatogonia (C) in the varicocele group.

Two months after varicocele induction, we investigated testis tissue sections from all three groups in this study. Figure-2A represents the deleterious effect of varicocele on testis structure. Our analysis showed that there is a significant reduction in the seminiferous tubules diameter following varicocele (190.51±19.23) (p=<0.001) (Figure-2B). In line with this finding, the number of spermatogonial decreased in the varicocele group (43.63±5.31) in comparision to the control (71.71±6.72) and sham groups (66.25±6) (p<0.001) (Figure-2C). Spermatogonial cells are found near the basement with dark nuclei. 

**Varicocele downregulates** ***CatSper*** **1 and 2**

**Figure 3:**
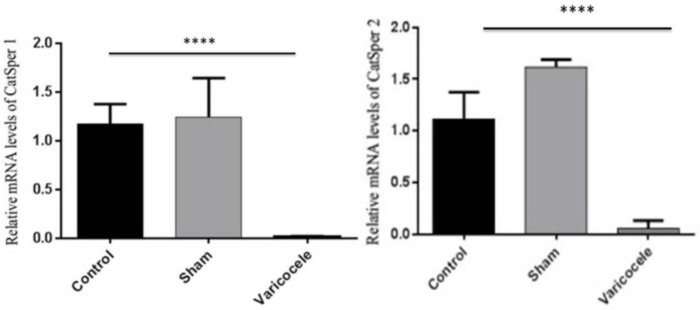
Expression pattern of *CatSper* 1 (A) and *CatSper* 2 (B) two months after varicocele induction was analyzed by real-time PCR. Levels of *CatSper* 1 and 2 were significantly decreased in the varicocele group compared to sham and control animals.

To verify the effects of partial ligation of the left renal vein on *CatSper* channels, we analyzed testis tissue for gene expression of *CatSper* 1 and 2 using real-time PCR. As we would expect, experimental varicocele induced a significant downregulation of *CatSper* 1 and 2 gene expression 2 months after varicocele induction. The mRNA level of both *CatSper* 1 and 2 was significantly lower in the varicocele group compared with the control and sham-operated animals (p<0.001) (Figure-3). 

**: p≤0.01

***: p≤0.001.

****: p≤0.0001, bar represents 50 µm.

****: p≤0.0001.

## Discussion

CatSpers (1-4) is a group of Ca2+ channels which have an important role in sperm motility (29). These channels are located in the principal piece of sperm flagellum (18) and were detected in the testis tissue 3 wk after birth (30). CatSper 1 and 2 have an essential role in normal male fertility (31, 32) and CatSper 3 and 4 are important in the acrosome reaction (33). 

The ability to penetrate zona pellucida in CatSper knockout sperm decreases (18, 19) and in human, a mutation in CatSper 1 and 2 leads asthenoteratozoospermia (34). According to our results from real-time PCR, there was a significant downregulation of *CatSper* 1 and 2 genes in the left experimental varicocele rats. Although Western blot was not performed, it is possible that *CatSper* expression reduces in the varicocele-induced animal. 

Rezaian co-workers showed that the expression of *CatSper*s 1 and 2 genes decreased 2 wk after spinal cord injury induction; however, *CatSper*s 3 and 4 showed no changes. They believed that downregulation in *CatSper* 1 and 2 gene expression is one of the causes of sperm motility reduction in the SCI mouse model (21). Amini co-workers suggested that Krack had deleterious effects on testis structure, sperm parameters and *CatSper* 1 and 2 gene expression (23). On the other hand, Selenium (an antioxidant) could up-regulate *CatSper* genes in the aging male mice (35). Mannowetz *et al* believed that pregnenolone sulfate like progesterone activates *CatSper* and sperm motility, but pristimerin and lupeol (plant triterpenoids) can decrease *CatSper* activity and prevent fertilization (36). So these channels might be a promising target for male contraception. The low expression of *CatSper* genes probably is not the only factor involved in decreasing sperm motility in varicocelized rats and other factors such as alteration in coenzyme Q10 (37, 38) and increase the level of antisperm antibody might be affected as well (39, 40). 

In addition of *CatSper* gene expression, we investigated sperm parameters and testis structure 2 month after left renal vein ligation in rats. Our data showed that all sperm parameters except sperm number reduced in the varicocele group. In the line of these results, Pasqualotto co-workers claimed that the infertile patients with varicocele have small testis, low sperm motility and count as well as high level of follicle stimulating hormone (41). The other result of this study was decreasing in the number of spermatogonia and diameter of seminiferous tubules in the varicocele group. Shiraishi co-workers believed that proliferating cell nuclear antigen expression which has an important role in DNA synthesis, decreases in infertile men with varicocele (42). In addition, Barqawi co-workers showed that 14 days after varicocele induction germ cell apoptosis increases (43). Therefore, this decrease in a number of spermatogonial and in diameter of seminiferous tubules might be because of the apoptosis induction following varicocele.

## Conclusion

In conclusion, our study showed that two members of CatSper family (CatSper 1 and 2) are downregulated in varicocele animal model. This finding might be a valuable tool for next studies to understand molecular mechanisms involved in reducing sperm motility in varicocele patients.
